# Dying to Defend: Neutrophil Death Pathways and their Implications in Immunity

**DOI:** 10.1002/advs.202306457

**Published:** 2023-12-03

**Authors:** Haiyue Tu, Haoyu Ren, Junjie Jiang, Changshun Shao, Yufang Shi, Peishan Li

**Affiliations:** ^1^ The First Affiliated Hospital of Soochow University State Key Laboratory of Radiation Medicine and Protection Institutes for Translational Medicine Suzhou Medical College of Soochow University Suzhou Jiangsu 215123 China

**Keywords:** ferroptosis, necroptosis, NETosis, neutrophil apoptosis, pyroptosis

## Abstract

Neutrophils, accounting for ≈70% of human peripheral leukocytes, are key cells countering bacterial and fungal infections. Neutrophil homeostasis involves a balance between cell maturation, migration, aging, and eventual death. Neutrophils undergo different death pathways depending on their interactions with microbes and external environmental cues. Neutrophil death has significant physiological implications and leads to distinct immunological outcomes. This review discusses the multifarious neutrophil death pathways, including apoptosis, NETosis, pyroptosis, necroptosis, and ferroptosis, and outlines their effects on immune responses and disease progression. Understanding the multifaceted aspects of neutrophil death, the intersections among signaling pathways and ramifications of immunity will help facilitate the development of novel therapeutic methods.

## Graphical abstract

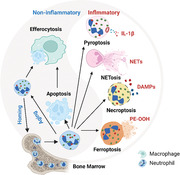

## 1 Introduction: Neutrophil Homeostasis and Death

Neutrophils, also known as polymorphonuclear leukocytes, are highly abundant in the circulation, accounting for up to 70% and 10–25% of all peripheral blood leukocytes in humans and mice, respectively.^[^ ^1^, ^2^ ^]^ They serve as primary defenders against bacterial and fungal pathogens. Their abundance also has clinical relevance. For example, diminished neutrophil counts due to inherent anomalies or acquired disorders often lead to severe infections.^[^ ^3^, ^4^ ^]^ However, their robust antimicrobial actions can act as a double‐edged sword, if unchecked, potentially exerting harmful effects on host tissues and leading to self‐inflicted diseases.^[^ ^5^ ^]^ Under physiological conditions, neutrophil demise and replacement are meticulously balanced via host‐driven mechanisms and microbial factors. Disruption of this delicate balance substantially alters the number and efficacy of neutrophils.

Mature neutrophils do not undergo cell division, possess lobulated nuclei, and have relatively short lifespans. Their continual replacement occurs in the bone marrow via granulopoiesis. Under steady‐state conditions, humans generate ≈1 billion neutrophils daily for every kilogram of body weight, and this number can increase to 10 billion in inflammatory cases.^[^ ^6^ ^]^ Granulopoiesis relies on the cytokine granulocyte colony‐stimulating factor. After entering the bloodstream, mature neutrophils have a half‐life of less than 24 h.^[^ ^7^ ^]^ Interestingly, granulopoiesis appears to depend on the phagocytosis of apoptotic neutrophils by tissue dendritic cells and macrophages. As apoptotic neutrophils become engulfed, a subsequent decline in interleukin‐23 (IL‐23) and IL‐17 levels is observed. This reduction curtails the production of granulocyte colony‐stimulating factor, thereby affecting the production of neutrophils.^[^ ^8^ ^]^

**Figure 1:**
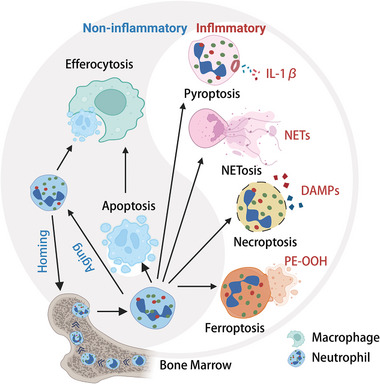
Neutrophil Homeostasis and Multifaceted Death Pathways in Steady‐State and Inflammatory Conditions. Neutrophils are generated in the bone marrow through granulopoiesis and subsequently enter the circulatory system. Depending on the specific microenvironment, neutrophils undergo various mechanisms of cell death.^[^ ^7^ ^]^ These mechanisms encompass both non‐lytic apoptosis and lytic death modalities, including necroptosis, pyroptosis, ferroptosis, and NETosis. These lytic processes are accompanied by the release of cytotoxic cellular proteases, cell‐free DNA, and chromatin into the microenvironment. Each death pathway operates through distinct molecular mechanisms and regulatory networks, ultimately resulting in either immunosuppressive or pro‐inflammatory outcomes.^[^ ^10^ ^]^ Defects in the clearance of apoptotic neutrophils and the accumulation of cellular remnants contribute to the onset of inflammatory diseases and autoimmune disorders. Figure created with BioRender.com.

Neutrophil homeostasis depends on a finely tuned balance among cell maturation, release from the bone marrow, migration through vascular and tissue channels, aging, and death.^[^ ^7^ ^]^ This trajectory is determined by factors ranging from inherent diurnal cell rhythms, interactions with both commensal and pathogenic microbes, environmental exposure to cellular senescence. Neutrophils can undergo various death modalities, such as apoptosis, NETosis, pyroptosis, necroptosis, and ferroptosis. Each modality distinctly modulates the immune responses and disease resolution pathways. Given the large cytotoxic repertoire of neutrophils, rigorous regulation of their production and clearance is necessary to avert unintended inflammatory repercussions in the host. Under physiological conditions, apoptotic neutrophil death modalities prevail, and apoptotic cells exert immunosuppressive effects by regulating T cell activation or phagocytosis by macrophages/dendritic cells.^[^ ^9^ ^]^ However, certain infectious or inflammatory stimuli induce the lytic neutrophil death pathways, resulting in the release of damage‐associated molecular patterns (DAMPs) and expulsion of cytotoxic granules (**Figure** **1**).^[^ ^10^ ^]^ When dysregulated, this process aggravates the localized inflammatory responses and propagates the injury to adjacent tissues. A comprehensive understanding of the intricacies of neutrophil death pathways and the distinctions and intersections among various cell death patterns is imperative for the development of precise therapeutic approaches for associated diseases. In this review, we discuss the different modes of neutrophil death, emphasizing their key roles in host antimicrobial and inflammatory responses, with special attention to the convergent and divergent signaling mechanisms and biological outcomes of each death modality.

## 2 Neutrophil Apoptosis

Apoptosis plays a key role in orchestrating neutrophil homeostasis and regulating inflammation. As neutrophils mature and migrate to peripheral tissues, apoptosis is a crucial in ensuring the equilibrium of circulating neutrophils and facilitating the efficient resolution of inflammatory responses.^[^ ^11^ ^]^

### 2.1 Molecular Mechanisms and Regulatory Pathways Governing Neutrophil Apoptosis

Apoptotic neutrophils exhibit distinctive morphological attributes. The alteration in cellular structure results in a reduced size, imparting a nearly spherical shape to the cells. This transformation is concomitant with or promptly followed by an extended phase of dynamic plasma membrane vesiculation,^[^ ^12^ ^]^ wherein phosphatidylserine translocates from the inner leaflet to the outer leaflet of the plasma membrane. Simultaneously, the chromatin undergoes condensation, leading to DNA fragmentation within the cell. Concomitantly, discernible shifts in organelle morphology occur.^[^ ^13^ ^]^ The mitochondria transition from a tubular network to a clustered configuration is accompanied by depolarization, and a multitude of vacuoles emerge within the cytoplasm.^[^ ^14^, ^15^ ^]^ Ultimately, these cells give rise to apoptotic bodies, which are subsequently engulfed and cleared by phagocytes.

**Figure 2:**
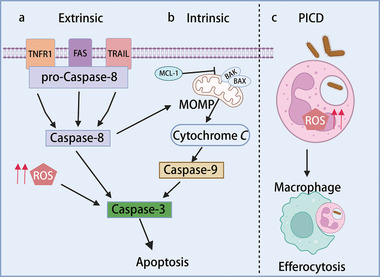
Molecular Mechanisms Underlying Neutrophil Apoptosis. a) **Extrinsic Apoptosis**: Initiated by cell surface death receptors, such as FAS, TNFR1, and TRAIL receptors, the extrinsic apoptotic pathway begins with the activation of Caspase‐8, which also promotes Mitochondrial Outer Membrane Permeabilization (MOMP), ultimately leading to Caspase‐3 activation, responsible for the execution phase.^[^ ^18^ ^]^ Concurrently, the generation of reactive oxygen species (ROS) by NADPH oxidase serves as a complementary factor in this pathway.^[^ ^19^ ^]^ b) **I** **ntrinsic Apoptosis**: Within the intrinsic apoptosis pathway, pro‐apoptotic dimers from the Bcl‐2 family, Bax and Bak, embed themselves in the mitochondrial outer membrane, inducing MOMP. The release of Cytochrome *c* into the cytoplasm then initiates the activation of Caspase‐9, eventually leading to Caspase‐3‐mediated apoptosis.^[^ ^16^ ^]^ c) **Pathogen‐Induced Cell Death (PICD)**: This specialized process integrates the phagocytic elimination of microbial pathogens with the initiation of apoptosis. Following their antimicrobial actions, neutrophils are targeted for clearance via efferocytosis, typically carried out by macrophages. Effective efferocytosis ensures the timely removal of apoptotic neutrophils, preventing their progression to secondary necrosis. Inefficient clearance of apoptotic neutrophils may lead to the extracellular release of toxic granules and damage‐associated molecular patterns (DAMPs), thus amplifying local inflammatory responses and perpetuating tissue injury.^[^ ^29^ ^]^ Figure created with BioRender.com.

Similar to other cell types, neutrophil apoptosis is initiated via two distinct signaling pathways, intrinsic and extrinsic (**Figure** **2**).^[^ ^16^ ^]^ Within the intrinsic pathway of apoptosis, pro‐apoptotic dimers of the Bcl‐2 family, specifically Bax and Bak, are embedded in the mitochondrial outer membrane. This action results in the induction of mitochondrial outer membrane permeabilization (MOMP), leading to the dissipation of the mitochondrial outer membrane potential. Consequently, cytochrome *c* is released into the cytoplasm, which initiates the activation of Caspase‐9. Subsequently, Caspase‐9 drives the activation of Caspase‐3, which is the central effector of the apoptotic process (Figure 2b).^[^ ^15^, ^17^ ^]^ Conversely, the extrinsic pathways are activated via the engagement of cell surface death receptors, such as FAS, tumor necrosis factor (TNF)‐receptor 1 (R1), and TNF‐associated apoptosis‐inducing ligand receptors, from the TNF receptor superfamily. This engagement activates Caspase‐8, which promotes MOMP, ultimately leading to the activation of Caspase‐3 responsible for the execution phase (Figure 2a).^[^ ^18^ ^]^ Exogenous apoptosis pathways in neutrophils rely heavily on reactive oxygen species (ROS) derived from NADPH oxidation. This has been corroborated using neutrophils isolated from patients with chronic granulomatous disease, in which a mutation at the *NOX2* locus impedes typical NOX‐mediated ROS production.^[^ ^19^ ^]^

Specific intrinsic factors render the neutrophils susceptible to apoptosis. Maturation of neutrophils decreases the transcription and translation rates, consequently diminishing the expression of anti‐apoptotic proteins though the expression of apoptotic effector proteins is preserved.^[^ ^20^ ^]^ Apoptosis is regulated by various factors, such as the mitochondrial apoptosis‐inducing factors.^[^ ^21^, ^22^ ^]^ Neutrophils are prolific generators of ROS.^[^ ^23^ ^]^ Their distinctive granulocytic system, which houses abundant proteases that activate Caspases, contributes to the initiation of apoptosis.^[^ ^24^, ^25^ ^]^ The intricate molecular signaling underlying spontaneous neutrophil apoptosis necessitates further investigation to unravel its origin. Unveiling these diverse molecular mechanisms can provide insights into the intricate balance governing neutrophil survival and death.

### 2.2 Pathogen‐Induced Neutrophil Apoptosis, Efferocytosis, and their Implications in Inflammation and Disease

In addition to classical death receptors, neutrophil apoptosis is also induced via pathogen‐induced cell death, a significant form of neutrophil apoptosis that intertwines microorganism ingestion and killing with apoptosis induction (Figure 2c).^[^ ^26^, ^27^ ^]^ Specialized phagocytes effectively clear neutrophils via efferocytosis.^[^ ^28^ ^]^ Ineffectual removal of apoptotic neutrophils leads to cell necrosis and subsequent extracellular release of toxic granulocytes and DAMPs, aggravating the local inflammatory responses and perpetuating tissue damage.^[^ ^29^ ^]^ Pathogens such as *Chlamydia pneumoniae* and *Klebsiella pneumoniae* hinder apoptosis and support their survival and replication by obstructing neutrophil apoptosis and efferocytosis.^[^ ^30^, ^31^ ^]^ Similar to death receptor‐induced neutrophil apoptosis, pathogen‐induced cell death relies on ROS production, as illustrated by chronic granulomatosis in which certain bacterial and fungal infections persist.^[^ ^32^ ^]^

Neutrophil efferocytosis deficiency contributes to the pathogenesis of atherosclerosis in humans. Accumulation of oxidized lipoproteins and atherogenic factors leads to an influx of leukocytes into the inflamed sites.^[^ ^33^ ^]^ Oxidized lipoproteins impair c‐Mer tyrosine kinase (MerTK)‐mediated macrophage receptor efferocytosis, thereby hampering the clearance of damaged apoptotic cells.^[^ ^34^ ^]^ Failure to curb inflammation causes macrophages to transform into “foam cells,” releasing pro‐inflammatory cytokines and attracting more neutrophils, culminating in a highly inflammatory “necrotic core,” characteristic of advanced atherosclerotic plaques.^[^ ^35^ ^]^

In summary, efferocytosis is a vital pathway for clearance of apoptotic neutrophil, fostering the regression of inflammation in inflammation‐related ailments. Pathogen‐induced cell death is beneficial for resolving acute inflammation and infection. Pathogen survival is partly attributed to delays in neutrophil apoptosis and cell lysis. Prolonged neutrophil survival is linked to diverse inflammatory and immune diseases, such as the systemic inflammatory response syndrome and autoimmune and rheumatic diseases.^[^ ^36^, ^37^ ^]^ Enhanced understanding of the molecular mechanisms governing neutrophil apoptosis is imperative to identify novel therapeutic targets and develop strategies to address pathologies associated with the dysregulation of neutrophil apoptosis.

### 2.3 Neutrophil Apoptosis‐Targeting Therapeutic Strategies for Inflammatory Diseases

Neutrophil apoptosis is a key mechanism in maintaining neutrophil homeostasis. Deficiencies in neutrophil apoptosis are associated with various inflammatory diseases and significantly influence the prognosis of infectious diseases. For instance, increasing alveolar neutrophil apoptosis mitigates inflammation and expedites clinical stability in patients with community‐acquired pneumonia.^[^ ^38^ ^]^

Neutrophil apoptosis is triggered by several mechanisms, including growth factor deprivation, death ligand binding, ROS generation, and pathogen‐induced events. Key regulators inhibiting neutrophil apoptosis include members of the BCL‐2 family and inhibitors of the apoptosis protein (IAP) family.^[^ ^39^ ^]^ Within the realm of apoptosis regulated by BCL‐2, members of the BCL‐2 family predominantly oversee the mitochondrial outer membrane permeabilization via intricate protein interactions. These members share a hydrophobic BH3 domain that is crucial for mediating these interactions. Interplay between BH3 and anti‐apoptotic BCL‐2 proteins serves as the foundation for the development of small‐molecule inhibitors mimicking BH3 protein‐binding properties. BH3‐only protein BIM provides relief in arthritis models, primarily by inducing apoptosis in macrophages and Gr1‐positive bone marrow cells.^[^ ^40^ ^]^

Specific MCL‐1 inhibitors show promising potential in arthritis models due to their role in promoting neutrophil apoptosis. Strategies targeting MCL‐1 and BCL‐XL, including use of inhibitor R‐roscovitine, a cyclin‐dependent kinase (CDK) inhibitor that reduces MCL‐1 protein levels and triggers neutrophil apoptosis, have successfully ameliorated arthritis in mouse models.^[^ ^41^, ^42^ ^]^ Neutrophils exhibit high sensitivity to FAS ligand (FASL)‐induced apoptosis and elevated levels of TNF‐α. However, findings regarding TRAIL are still a subject of debate. Clinically approved human intravenous immunoglobulin (IVIg) formulations have been demonstrated to induce neutrophil apoptosis, with reactive anti‐FAS antibodies identified as the active components.^[^ ^43^ ^]^ Moreover, stimulatory anti‐Siglec‐9 antibodies found in IVIg formulations have been shown to induce neutrophil death via both apoptotic and non‐apoptotic pathways.^[^ ^44^ ^]^ Notably, the IVIg preparations did not induce cell death in mouse neutrophils in the same manner as that in humans, highlighting a significant interspecies difference.^[^ ^45^ ^]^

Furthermore, advancements in nanotechnology and biotechnology have paved the way for innovative approaches targeting the specific treatment of pro‐inflammatory neutrophils for the management of inflammatory diseases. The novel strategy of nanoparticles targeting activated inflammatory neutrophils for the delivery of pro‐apoptotic drugs offers an alternative to the systemic inhibition associated with current anti‐inflammatory medications.^[^ ^46^ ^]^

## 3 NETosis

**Figure 3:**
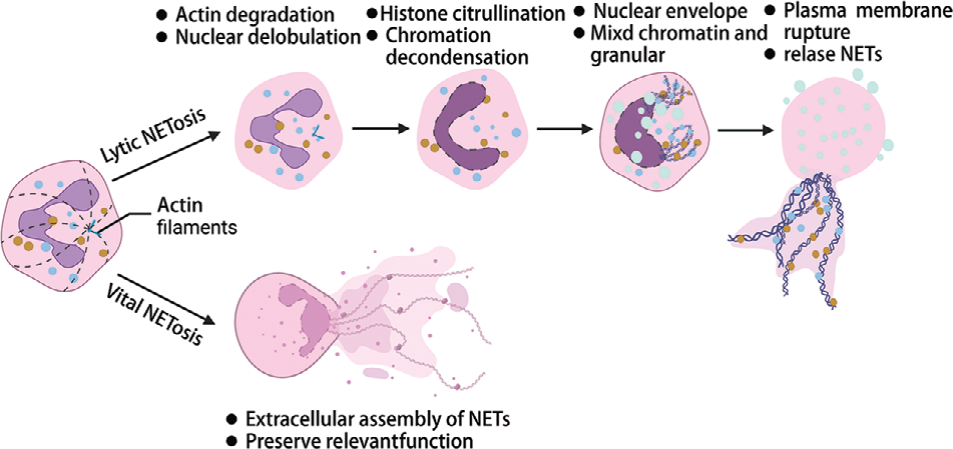
Pathways of Neutrophil Extracellular Trap (NET) Formation. a) **Lytic NETosis**: This form of NET formation is characterized as a cell death pathway. It commences with the disassembly of actin cytoskeletal structures within the neutrophil, followed by nuclear delobulation, which involves the reorganization of nuclear components.^[^ ^47^ ^]^ Subsequent histone citrullination facilitates chromatin decondensation, allowing de‐agglutinated chromatin to mix with cytoplasmic granular components. The process culminates in plasma membrane rupture, releasing NETs into the extracellular milieu.^[^ ^50^ ^]^ b) **Vital NETosis (Non‐Lytic NETosis)**: In contrast, vital NETosis enables neutrophils to form NETs without accompanying cell death.^[^ ^48^ ^]^ During this process, NETs are extruded from neutrophils while maintaining membrane integrity. This non‐lytic mode of NET formation maintains the neutrophil's functional capacity for tasks such as phagocytosis, allowing them to engage in microorganism engulfment and contribute to host defense while simultaneously releasing NETs.^[^ ^49^ ^]^ Figure created with BioRender.com.

NETosis, a distinctive form of regulated cell death, remained obscure until 2004, when its crucial role in neutrophil function was revealed. Brinkmann et al. showed that neutrophils release chromatin loaded with granular antimicrobial proteins.^[^ ^47^ ^]^ This process not only restricts bacterial dissemination by enhancing their adhesion within host cells but also enables fibrous chromatin structures to neutralize virulence factors and eliminate bacteria, augmenting the innate immune system defenses against acute infections. These fibrous extracellular constructs, now called neutrophil extracellular traps (NETs), were initially linked to cell death and the term NETosis was coined. Notably, not every pathway leading to NET formation results in cell death, even under controlled in vitro conditions, which has fueled the debate over the scope of NETosis. Two distinct forms of NETosis have been identified: classical or suicidal NETosis, which leads to cell death, and “vital NETosis,” which maintains cellular viability along with a range of effector functions (**Figure** **3**).^[^ ^48^, ^49^ ^]^

### 3.1 Complexity of NETosis: Mechanisms, Triggers, and Pathophysiological Implications

NETosis involves a series of morphological alterations in neutrophils, such as arrested actin dynamics, depolarization, and loss of nuclear lobules. Subsequently, histone citrullination leads to chromatin decondensation. Consequently, the nuclear membrane disintegrates into vesicles containing dispersed chromatin, resulting in numerous vacuoles within the cells.^[^ ^50^ ^]^ These vacuoles differ from the cellular blistering observed during pyroptosis, which originates from the nuclear envelope that contains condensed chromatin.^[^ ^51^ ^]^ Then, breakdown of the nuclear vesicles and cytoplasmic particles occurs, enabling the de‐agglutinated chromatin to mix with cytoplasmic granular components, such as lactoferrin, BPI, LL‐37, and histones. These elements are crucial for the formation of NETs that trap and eliminate bacteria.^[^ ^52^, ^53^ ^]^ Ultimately, the plasma membrane disintegrates, releasing chromatin into the extracellular space, where it forms extracellular traps known as NETs (Figure 3). This process enables the neutrophils to continue their bactericidal function beyond their limited lifespan, thereby extending their antimicrobial activity.

**Figure 4:**
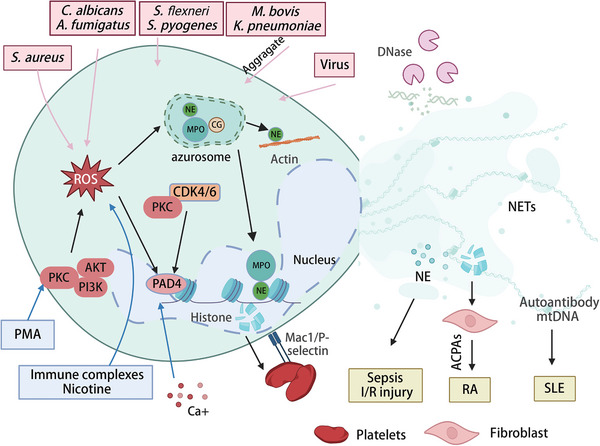
Regulatory Mechanism of NET Formation and Release. The generation of ROS serves as a cornerstone in modulating NET release. Key kinases such as AKT, PI3K, and PKC are integral to the dynamic regulation of ROS levels.^[^ ^63^, ^64^ ^]^ Elevated intracellular calcium ions are indispensable for NETosis and act as activators for PAD4, which in turn catalyzes histone citrullination and chromatin decondensation.^[^ ^52^ ^]^ In synergy with PKC and CDK4/6, PAD4 facilitates the disassembly of nuclear architecture during NETosis.^[^ ^70^ ^]^ The azurosome, a specialized organelle, harbors key enzymes like Myeloperoxidase (MPO), Neutrophil Elastase (NE), and Cathepsin G (CG), essential for NET biogenesis. Upon neutrophil activation, these enzymes translocate to the nucleus, furthering chromatin relaxation. The release of NE into the cytosol is ROS‐ and MPO‐mediated and precedes its nuclear translocation, wherein it targets and degrades F‐actin. Both pathogens and Pathogen‐Associated Molecular Patterns are potent NET inducers.^[^ ^69^ ^]^ Dysregulated NET formation or defective clearance can result in pathological NET accumulation, exacerbating inflammation and autoimmune diseases. Furthermore, NETs can interact with platelets, presenting potential complications such as vascular or catheter obstructions.^[^ ^111^ ^]^ This figure serves as a comprehensive synopsis of the multifaceted processes underpinning NET formation, underscoring its relevance in host defense, inflammatory regulation, and disease etiology. ACPAs, autoantibodies to citrullinated protein antigens; I/R injury, ischemia‐reperfusion injury; RA, rheumatoid arthritis; SLE, Systemic Lupus Erythematosus. Figure created with BioRender.com.

NETosis can be induced by many factors, including pathogens, lipopolysaccharides (LPS), antibodies, immune complexes, specific cytokines such as IL‐8 and TNF‐α,^[^ ^54^, ^55^ ^]^ complement C3,^[^ ^56^ ^]^ cholesterol crystals,^[^ ^57^ ^]^ microcrystals,^[^ ^58^ ^]^ and drugs such as phorbol myristate acetate (PMA), calcium, and potassium ionophores. Notably, the presence of NETs has been observed in vivo in experimental dysentery and spontaneous human appendicitis^[^ ^59^ ^]^ underscoring that NETs are more than a consequence of PMA toxicity and represent a potentially relevant physiological process. Furthermore, the release is generally regarded as an active cellular process initiated by intrinsic pathways activated by external stimuli.^[^ ^60^ ^]^ These findings implicate NETs in various physiological processes (**Figure** **4**).

Initially, many studies investigating the molecular mechanisms of NETs focused on induction model utilizing PMA. This compound directly activates protein kinase C (PKC),^[^ ^61^ ^]^ leading to the phosphorylation of NADPH oxidase (NOX2) and the resultant generation of ROS.^[^ ^62^ ^]^ Downstream of PKC, a multitude of kinases are activated, including c‐Raf, MEK, PI3K‐Akt, and ERK.^[^ ^63^, ^64^ ^]^ Nonetheless, the precise function of these kinases remains unclear, with conflicting evidence. It is plausible that the overlap of downstream PKC signals, which can directly activate NADPH oxidase, contributes to this discrepancy. Exploration of the roles of these kinases in inducing NETosis has also highlighted the involvement of autophagy in the release of NETs. PI3K is indispensable for both NETosis and autophagy. NET release is hindered in the absence of ATG5/ATG7, essential autophagy proteins.^[^ ^65^, ^66^ ^]^

Activation of NETosis depends on NOX2‐mediated ROS production, which triggers Myeloperoxidase (MPO) activation (Figure 4). This activation initiates the translocation of neutrophil elastase (NE) from the azurosome to the nucleus. In the nucleus, NE aids histone proteolysis and disrupts chromatin packaging. Subsequently, MPO binds to chromatin and collaborates with NE to condense the chromatin, independent of its enzymatic activity.^[^ ^67^ ^]^ A segment of MPO binds to NE, forming a complex called the azurosome. This complex liberates NE from the azurosome particles via hydrogen peroxide.^[^ ^68^ ^]^ Subsequently, NE is released into the cytoplasm where it binds to F‐actin filaments, undergoes degradation, and enters the nucleus.^[^ ^69^ ^]^ Moreover, NE may contribute to nuclear membrane degradation by promoting the release of chromatin into the cytoplasm. This process involves the fusion of various granular contents with de‐agglutinated chromatin, plasma membrane ruptures, and NETs into the extracellular space. The introduction of exogenous ROS to the neutrophils of patients with chronic granulomatosis can stimulate NET formation, confirming the important role of ROS in NET formation.^[^ ^50^ ^]^

In addition to NOX2, activation of cyclin‐dependent kinases (CDK), which contribute to cell cycle entry, can trigger NETosis.^[^ ^70^ ^]^ CDK4/6 and PKC mediate the disruption of the structural rigidity of the nucleus governed by the lamin A network, releasing chromatin during NETosis (Figure 4). Non‐NADPH oxidation‐dependent triggers of NETosis include immune complexes and nicotine. These triggers rely on the production of mitochondrial ROS.^[^ ^71^, ^72^ ^]^ Furthermore, vital nonlytic NET production is believed to occur independently of ROS.^[^ ^48^, ^49^ ^]^ Therefore, different pathological stimuli may induce NETs via distinct pathways.

Histone citrullination and its proteolytic cleavage are considered essential for chromatin disassembly and the subsequent release of DNA during NETosis. One enzyme that plays a key role in this process is peptidylarginine deiminase 4 (PAD4), which removes positive charges from histones by converting arginine residues into citrulline (Figure 4). This conversion results in chromatin decondensation, ultimately leading to rupture of the nuclear envelope.^[^ ^52^, ^73^ ^]^ The involvement of PAD4 in NET formation remains controversial. In light of calcium ionophore‐induced NETosis, PAD4 has been proposed as a Ca^2+^‐activated enzyme responsible for histone citrullination, serving as a link between ROS production and chromatin decondensation during NETosis.^[^ ^52^ ^]^ Inhibition of PAD4 has been shown reduces NET formation in response to specific stimuli. Notably, *PAD4*‐deficient mice exhibit impaired NET formation in some cases.^[^ ^73^, ^74^, ^75^ ^]^ However, other studies have indicated that PMA‐induced NETs formation is unaffected by PAD4 inhibition.^[^ ^59^ ^]^ This suggests that PAD4 is not the sole determinant of NET formation. These variations in the role of PAD4 in NET formation may stem from our limited understanding of the diverse functions of PAD4 in neutrophil biology. Studies focusing on the role of citrullination in NETosis, particularly those induced by ionophores, have primarily focused on the analysis of histone proteins.^[^ ^71^ ^]^ Interestingly, calcium ionophores induce extensive protein citrullination within cells. Beyond its role in promoting protein citrullination, the catalytic activity of PAD4 hinders the assembly and activation of NOX2.^[^ ^76^ ^]^ Therefore, when PAD4 is considered a driving factor for NETosis, its function must be assessed with multiple detection methods.

### 3.2 NETosis in Host–Pathogen Interactions: Molecular Mechanisms, Pathogen Counterstrategies, and Clinical Implications

NETosis, a crucial mechanism for combating fungal and bacterial infections, uses NETs to entrap and eliminate pathogens directly via granule‐derived proteins. Early foundational studies have established that *Staphylococcus aureus* triggers ROS‐induced NETosis through NOX2.^[^ ^50^ ^]^ As a result, neutrophil in patients with chronic granulomatous disease are unable to undergo NETosis in response to *Staphylococcus aureus* or PMA.^[^ ^77^ ^]^ However, revolutionary approaches, such as gene therapy and the introduction of exogenous ROS, are capable of inducing NET formation and restore antibacterial efficacy.^[^ ^50^, ^77^ ^]^ Remarkably, in a mouse model of *Staphylococcus aureus* skin infection, prompt release of NETs by neutrophils effectively thwarts systemic bacterial transmission via the bloodstream.^[^ ^50^ ^]^

*Staphylococcus aureus* – induced NET release occurs through the NADPH oxidation‐independent pathway, signifying the capacity of neutrophils to sustain transient function while navigating toward the infection site, characterized as the non‐lytic form of NETosis.^[^ ^78^ ^]^ The mechanisms by which bacteria induce NET formation vary between studies. NETosis is triggered by *Candida albicans* and *Aspergillus fumigatus* through the action of NADPH oxidase, MPO, and NE. ^[^ ^79^, ^80^, ^81^ ^]^ Neutrophils can discern the size of microorganisms, prompting NETosis in response to larger pathogens, such as *Candida albicans* mycelia and bacterial aggregates.^[^ ^80^ ^]^ Conversely, smaller microorganisms are engulfed by intracellular phagosomes and undergo fusion with azurosomes. This suppresses NE expression and inhibits chromatin condensation.^[^ ^69^, ^80^ ^]^ Selective induction of NETosis serves as the key mechanism for mitigating superfluous tissue damage in response to infections.

However, several small extracellular and intracellular pathogens, including viruses, can induce neurotoxicity.^[^ ^82^ ^]^ Many of these microorganisms have evolved mechanisms to survive and evade phagosomes. Consequently, NETosis may be reserved for smaller microorganisms that impede phagosome‐mediated killing. *Neisseria gonorrhoeae*, for instance, disrupts phagosome and azurosome fusion to impede its bactericidal effects, thereby triggering NETosis.^[^ ^83^ ^]^ Aggregation is a strategy used by smaller microbes to eliminate phagocytosis. Extensive aggregates of *Mycobacterium bovis* and clusters of *Bacillus Calmette‐Guérin* induce NETosis in a size‐dependent manner.^[^ ^67^, ^80^ ^]^ Taken together, microbe‐induced NETosis hinges on the size and pathogenicity of the microbes. This mechanism enhances our understanding of host defense strategies and offers insights into the fascinating interplay between microbial size and behavior and host immune responses.

NETs play pivotal roles in combating specific infections; however, the intricate molecular mechanisms underlying their antimicrobial efficacy remain unclear. Key molecules, such as NADPH oxidase, MPO, and PAD4, are integral to inducing NETosis. Notably, patients deficient in glucose 6‐phosphate dehydrogenase, the first enzyme of the oxidizing branch of the pentose phosphate pathway, exhibit diminished NADPH and ROS levels, making them more susceptible to bacterial infections.^[^ ^84^ ^]^ Additionally, MPO deficiency in humans is primarily linked to recurrent fungal infections,^[^ ^85^ ^]^ which is supported by similar findings in *MPO*‐deficient mice. This finding reinforces the key roles of NETs in fungal infections. In contrast, the influence of NETosis on host susceptibility to bacteria and survival in septic mice remains unchanged in *PAD4*‐deficient mice.^[^ ^86^ ^]^ Nonetheless, *PAD4*‐deficient neutrophils exhibit impaired antibacterial activity against pathogens, such as *Shigella flexneri* and group A *Streptococcus pyogenes*, consequently exacerbating necrotizing fasciitis.^[^ ^73^ ^]^ These investigations underscore the significance of NETs in treating virulent bacterial and fungal infections.

Extracellular trap formation is an ancient and evolutionarily conserved defense mechanism.^[^ ^87^ ^]^ However, bacteria have developed strategies to evade NETosis‐induced killing. Numerous microorganisms express endonucleases that effectively degrade NETs.^[^ ^88^ ^]^ *Pseudomonas aeruginosa* and group A *Streptococcus* suppress NETosis via Siglec‐9 by coating with host sialylated glycoproteins.^[^ ^89^, ^90^ ^]^ Similarly, group B *Streptococcus* deploys molecules resembling sialic acids to dampen the ROS burst and reduce NETosis.^[^ ^91^ ^]^ NET release has also been observed in response to viruses, such as the human immunodeficiency and respiratory syncytial viruses. In particular, HIV‐1 virions stimulate dendritic cells to generate IL‐10, thereby safeguarding the virus from NET‐related protease degradation.^[^ ^82^ ^]^ Additionally, the Hepatitis B virus exploits HBV E and HBV C proteins to curtail ROS production in neutrophils, effectively inhibiting NETosis.^[^ ^92^ ^]^ (Figure 4).

### 3.3 Janus‐Faced Role of NETs in Immune Regulation, Autoimmunity, and Thrombosis

In addition to their antimicrobial functions, NETs play pivotal roles in coordinating the adaptive immune microenvironment and modulating inflammatory cytokines as key regulators of the immune response.^[^ ^93^, ^94^ ^]^ The roles of NET components in tissue damage during aseptic disease have been widely reported. In addition to triggering immune system activation, NETs amplify the efficacy of antibacterial agents by concentrating them within the fiber network. This, in turn, mitigates the harm inflicted on host tissues upon exposure to these components.^[^ ^95^ ^]^ Another important part of the regulatory effect of NETs on inflammation is the degradation of cytokines and chemokines by various proteases present in NETs.^[^ ^96^, ^97^ ^]^ In mouse models of gout induced by monosodium urate crystals, inflammation and gouty arthritis are more pronounced in ROS‐deficient mice that are unable to induce NETs.^[^ ^96^ ^]^ This underscores the anti‐inflammatory properties of NETs. NETs also exert anti‐inflammatory effects by modulating various immune cells. For instance, they regulate the functioning of dendritic cells and orchestrate the inflammatory response by increasing the expression of TH2 cytokines, while dampening the synthesis of TH1 and TH17 cytokines.^[^ ^98^ ^]^

However, excessive NET formation due to dysregulation and impaired elimination mechanisms can lead to detrimental outcomes, such as inflammation, autoimmune pathology, and vascular or catheter obstruction (Figure 4). Sterile inflammatory conditions often lead to the release of NETs, which can promote thrombosis, intensify local tissue damage, result in organ failure, and even death.^[^ ^99^ ^]^ A notable example of organ dysfunction linked to excessive NETs formation is the rapid onset of acute respiratory distress syndrome (ARDS).^[^ ^100^ ^]^ In some conditions, such as sepsis and acute injury, mitigating NETosis by NE or PAD4 defection has been shown to alleviate liver injury in mice.^[^ ^101^ ^]^ Central to this is the role of NET‐bound histones in mediating cytotoxicity.^[^ ^102^ ^]^ These free histones are not only cytotoxic and damage cell membrane integrity, but also activate TLR2 and TLR4 within NETs.^[^ ^103^ ^]^ Furthermore, in a mouse model studying ischemia‐reperfusion injury, NETs exacerbated inflammation and liver damage. Interventions using DNase or PAD4 inhibitors effectively reduce inflammation and liver damage.^[^ ^104^ ^]^

Systemic lupus erythematosus (SLE) is an autoimmune disease characterized by spontaneous NETosis in low‐density neutrophils. NETs contain increased levels of autoantigens and mtDNA, which intensify the autoimmune and inflammatory processes.^[^ ^105^ ^]^ Rheumatoid arthritis, which is characterized by autoantibodies against citrullinated protein antigens, is characterized by externalized citrullinated autoantigens and immunostimulatory molecules. The pathogenesis of rheumatoid arthritis involves accelerated NETosis, with NETs exacerbating the inflammatory response, thereby amplifying disease progression.^[^ ^106^ ^]^ Additionally, in a rheumatoid arthritis model, fibro‐engulfed NETs prompted the generation of citrulline histone antibodies.^[^ ^107^ ^]^ Similar trends link NETosis to autoimmune conditions, such as psoriasis, type 1 diabetes, and small vessel vasculitis.^[^ ^108^, ^109^, ^110^ ^]^

NETosis plays a pivotal role in the pathogenesis of thrombosis. This serves as the foundation of thrombosis and is associated with an exaggerated innate immune response. Notably, the interplay between neutrophils and platelets contributes to thrombosis.^[^ ^111^ ^]^ When activated by stimuli, such as LPS, histones, and arachidonic acid, platelets can potently induce NETs. This induction can occur through direct interactions facilitated by the P‐Selectin‐Mac1 axis^[^ ^112^ ^]^ or via the release of soluble mediators, such as High‐mobility group protein B1, Thromboxane A2, Platelet factor 4, von Willebrand Factor, and CCL5.^[^ ^112^, ^113^ ^]^ Additionally, platelets can release oxidized mitochondrial DNA,^[^ ^114^ ^]^ which further stimulates NETosis.^[^ ^115^, ^116^ ^]^ An indicator of the progression of pathological injury in lung disease is heme release. Moreover, heme‐induced platelet activation not only enhances platelet‐neutrophil aggregation, but also leads to the accumulation of NETs within the lungs, thereby exacerbating lung injury. Notably, a significant reduction in the likelihood of thrombosis was observed in a PAD4 knockout mouse model with inferior vena cava stenosis.^[^ ^117^ ^]^

In conclusion, immune thrombosis serves as a protective response against the capture and elimination of pathogens. However, excessive accumulation of NETs, along with their interactions with platelets and endothelial cells, fuels the formation of disease cascades, culminating in the development of substantial aggregates. This, in turn, contributes to the genesis of atherosclerotic plaques and exacerbates the pathological processes associated with ischemic stroke and myocardial infarction.

## 4 Neutrophil Pyroptosis

In contrast to apoptosis, a non‐inflammatory process that encapsulates cellular contents within intact vesicles, pyroptosis is a unique lytic cell death pathway inextricably linked to inflammation. Pyroptosis is primarily driven by host defense mechanisms in response to external invasions or environmental stressors, with the overarching goals of promoting tissue repair and halting the spread of infection.^[^ ^118^ ^]^ Pyroptosis induces cell membrane rupture, leading to the release of cytosolic contents, notably the pro‐inflammatory cytokines IL‐1β and IL‐18. This exodus serves two purposes: an immediate defense against intracellular pathogens and a signal to attract additional immune cells to the site of infection or injury.^[^ ^119^ ^]^ Notably, neutrophils, as central immune players, are significant contributors of IL‐1β and other cytokines, attributed to their rapid mobilization to infection and inflammation sites.^[^ ^120^ ^]^

### 4.1 Neutrophil Pyroptosis: Molecular Mechanisms, Triggers, and Morphological Insights

**Figure 5:**
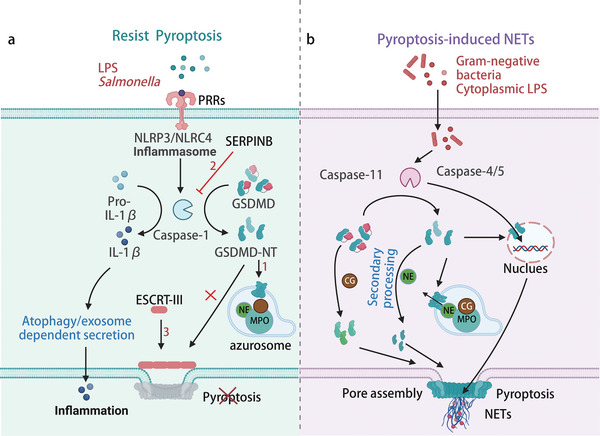
Potential Mechanisms of Neutrophil Pyroptosis. a) **Mechanisms Underpinning Resistance to Neutrophil Pyroptosis**: Pattern recognition receptors (PRRs), including NLRP3 and NLRC4, enable neutrophils to sense exogenous pathogens, culminating in inflammasome assembly and Caspase‐1 activation.^[^ ^126^ ^]^ In addition, this activation cascade results in the mature release of IL‐1β without invoking neutrophil pyroptosis.^[^ ^128^ ^]^ Several regulatory pathways contribute to this resistance: (1) neutrophil‐derived GSDMD‐NT targeting azurosomes and autophagosomes, limiting its impact on cell membranes. ^[^ ^129^ ^]^ (2) the serine protease inhibitors from the SERPINB family can impede inflammatory Caspases and neutrophil serine protease (NSP) activity, preventing unwarranted neutrophil pyroptosis.^[^ ^130^ ^]^ (3) the triggering of membrane repair mechanisms by GSDMD perforation, such as ESCRT‐III, which might support neutrophil resistance to GSDMD‐caused cytoplasmic membrane disruption.^[^ ^133^ ^]^ b) **Neutrophil Pyroptosis‐Induced NETs Pathway**: Intracytoplasmic LPS and bacterial agents activate Caspases, including Caspase‐11 and Caspase‐4/5, resulting in GSDMD‐dependent neutrophil death. These Caspases act in concert with GSDMD to facilitate nuclear membrane penetration and histone degradation, processes integral to NET formation.^[^ ^136^ ^]^ Neutrophil granules harbor specialized serine proteases, such as neutrophil elastase (NE) and cathepsin G (CTSG), capable of uniquely cleaving GSDMD, thus contributing to neutrophil pyroptosis.^[^ ^138^ ^]^ Figure created with BioRender.com.

In response to exogenous pathogens, such as LPS from Gram‐negative bacteria belonging to Pathogen‐Associated Molecular Patterns (PAMPs), or endogenous stimuli (e.g., cellular damage or stress‐induced release of DAMPs, such as ATP, uric acid, and nucleic acids), immune cells (such as neutrophils and macrophages) use Pattern Recognition Receptors (PRRs) to identify these molecular patterns. Activation of inflammasome assembly, commonly the canonical complexes involving nucleotide‐binding domain and leucine‐rich repeat receptor (NLR) or Absent in melanoma 2 (AIM2), adaptor protein ASC, and effector molecule Caspase‐1.^[^ ^121^ ^]^ Caspase‐1 processes the pro‐inflammatory cytokines, IL‐1β and IL‐18, releasing their mature forms (**Figure** **5**). A groundbreaking discovery in 2015 revealed that inflammatory Caspases (Caspase‐1, ‐4, ‐5, and ‐11) activate Gasdermin D (GSDMD), which perforates the cell membrane and initiates pyroptosis upon cleavage.^[^ ^122^ ^]^ GSDMD, comprising an ≈30 kDa amino‐terminal fragment (GSDMD‐NT) and approximately 20 kDa carboxy‐terminal fragment (GSDMD‐CT), maintains stability via the integration of GSDMD‐CT into the ring of GSDMD‐NT, thereby inhibiting GSDMD‐NT‐mediated pyroptosis.^[^ ^123^ ^]^

Morphologically, pyroptosis embodies characteristics of both necrosis and apoptosis. Unlike the abrupt disintegration observed in necrotic cell death, pyroptosis exhibits cytoplasmic flattening owing to leakage of the plasma membrane.^[^ ^124^ ^]^ During pyroptosis, perforation of the cell membrane by GSDMD leads to subtle cellular swelling. This is attributed to the influx of water into the cytoplasm, induced by intracellular non‐ionic penetrants. As a result, bubble‐like protrusions resembling apoptotic bodies and termed ″pyroptotic bodies emerged on the cell membrane. The cell then experiences pervasive pore formation in its membrane, leading to the loss of membrane integrity and chromatin damage. In the final stage, the membrane breaks down entirely, rendering the cytoplasmic vacuoles.^[^ ^125^ ^]^ The ensuing release of cellular contents acts as a catalyst for inflammation.

### 4.2 Neutrophil Inflammasome Activation and Pyroptosis: An Intricate Balance

Neutrophils are frontline defenders against infections and are rich in PRRs. However, induction of pyroptosis in neutrophils via inflammasome activation remains unclear. Several studies argue against Caspase‐1 instigating pyroptosis in neutrophils.^[^ ^126^, ^127^ ^]^ Instead, neutrophils appear to evade Caspase‐1‐mediated pyroptosis, ensuring sustained cytokine production at infection sites while maintaining their primary antimicrobial capabilities.^[^ ^126^ ^]^ In both human and mouse neutrophils, the TLR4 agonist LPS upregulates NLRP3 and NLRC4, leading to significant IL‐1β secretion. However, even after Caspase‐1 activation following inflammasome engagement, neutrophils don't undergo lytic death. Similar findings were observed in *Salmonella*‐infected mice.^[^ ^126^, ^128^ ^]^ GSDMD‐NT, the product of GSDMD cleavage, doesn't increase neutrophil membrane permeability or induce pyroptotic death. Potential reasons include: (1) neutrophil‐derived GSDMD‐NT targeting neutrophilic granules and autophagosomes, limiting its impact on cell membranes;^[^ ^129^ ^]^ (2) the necessity for stringent regulation of cell death, inflammation, and pyroptosis in neutrophils – molecules like the Serpin family B members (SERPINB) inhibit inflammatory Caspase and neutrophil serine protease (NSP) activity, preventing unwarranted neutrophil pyroptosis and ensuring their anti‐infection function;^[^ ^130^, ^131^ ^]^ (3) the triggering of membrane repair mechanisms by GSDMD perforation, such as ESCRT‐III, which might support neutrophil resistance to GSDMD‐caused cytoplasmic membrane perforation.^[^ ^132^, ^133^ ^]^ Notably, mature IL‐1β in neutrophils is found in plasma membrane infoldings, hinting at a GSDMD‐independent release mechanism.^[^ ^134^ ^]^ Additionally, cytokines may be released via autophagosome‐dependent secretion routes or via exosomes independent of GSDMD.^[^ ^135^ ^]^ This may account for the release of crucial inflammatory factors by neutrophils without undergoing pyroptosis.

In summary, although neutrophils do not completely block GSDMD‐induced membrane cleavage, as evidenced by azurosome membrane fragmentation, there is likely to be a regulatory mechanism governing GSDMD production and quantity. Exposure of neutrophils to cytoplasmic LPS or gram‐negative bacteria activates non‐canonical inflammasome (Caspase‐4/5/11) signaling and triggers GSDMD‐dependent neutrophil lysis.^[^ ^136^, ^137^ ^]^ Aging neutrophils induced NE‐dependent GSDMD cleavage and neutrophil death in vitro, validating GSDMD‐mediated plasma membrane fragmentation.^[^ ^138^ ^]^ Intriguingly, Caspase‐11 and GSDMD led to NET extrusion by mediating synergies between nuclear membrane permeabilization and histone degradation (Figure 5). Caspase‐11 activation propels NETosis in neutrophils exposed to monosodium urate and in those from septic mice and patients.^[^ ^139^, ^140^ ^]^ This underscores the potential interplay between neutrophil pyroptosis and NETosis, suggesting that GSDMD‐induced cytoplasmic membrane rupture may lead to NETs release.

### 4.3 Regulation of Neutrophil Pyroptosis: The Intricate Interplay between Granzymes and NSPs

The unique granzyme system of neutrophils significantly influences pyroptosis regulation via specific serine proteases capable of GSDMD activation in neutrophils. Elastase, an NSP within cytoplasmic particles, cleaves C268, which is seven residues upstream of D275 Caspase‐1 cleavage site in hGSDMD.^[^ ^138^ ^]^ This generates a functional GSDMD‐NT fragment that punctures the plasma membrane. The absence of Elastase or GSDMD prolongs the neutrophil lifespan, suggesting that Elastase and GSDMD activation induce pyroptosis in neutrophils.^[^ ^138^ ^]^ Another GSDMD‐cleaving NSP is Cathepsin G, which targets L274. Cleaved GSDMD triggers pyroptosis and releases inflammatory cytokines, such as IL‐1β, from neutrophils. Serpinb1 and Serpinb6 negatively regulate GSDMD cleavage by Cathepsin G, underscoring the strict control over neutrophil death pathways and cytokine release.^[^ ^130^, ^131^ ^]^ GSDMD‐NT can specifically target azurosome membranes while sparing other granular membranes, possibly due to affinity variations in distinct lipid structures. The dynamic evolution of the unique granular system of neutrophils during development suggests that developing neutrophils may be more susceptible to the regulation of pyroptosis. These insights suggest the existence of activation and inhibition events contingent on proteases and gasdermin proteins; however, the intricacies of neutrophil protease–gasdermin protein interactions remain unknown.

### 4.4 GSDMD in Neutrophil Death: Bridging Pyroptosis and NETosis in Immune Responses

GSDMD plays a multifaceted role in various neutrophil death pathways, including pyroptosis‐induced intracellular trap‐driven efferocytosis^[^ ^141^ ^]^ and NETosis. Pyroptosis and NETosis often occur during similar disease scenarios, necessitating immune responses against microbes or sterile inflammation. Exposure of neutrophils to cytoplasmic LPS or Gram‐negative bacteria activates non‐classical (Caspase‐4/11) inflammasomes, culminating in GSDMD‐dependent neutrophil death and NETs release. Caspase‐11 and GSDMD are pivotal for neutrophil plasma membrane rupture during the final stages of NET extrusion. Additionally, they facilitate early NETosis formation by mediating nuclear membrane penetration and histone degradation during DNA decondensation.^[^ ^136^ ^]^ Caspase‐11 is involved in NETs formation and has been validated in mouse models of Acute Gouty Arthritisand humans with sepsis.^[^ ^136^, ^139^ ^]^

GSDMD‐mediated NETosis is determined depending on the shear mode and disease context. NETs play dual roles in combating bacteria, while potentially inflicting tissue damage. Hence, the activation of GSDMD and the consequent NETs generation should be tightly regulated. Amidst numerous unresolved questions are which pathways dictate GSDMD fragment processing leading to cell lysis and which pathways are employed to fend off microbial attacks or sterile inflammation remain unknown.^[^ ^10^ ^]^ In summary, GSDMD‐mediated neutrophil death offers a distinct target for anti‐inflammatory and antimicrobial therapies, although further research is required to elucidate its precise role.

### 4.5 Neutrophil Pyroptosis in Inflammatory Diseases and Potential Therapeutic Targets

In normal physiological processes, pyroptosis plays a pivotal role in host defense against pathogenic infections. However, excessive pyroptosis can lead to an overwhelming and sustained inflammatory response that has been implicated in the pathogenesis of various inflammatory diseases. While the existing literature on pyroptosis has predominantly focused on monocytes and macrophages, recent studies have shed light on the significance of pyroptosis in neutrophils.

Neutrophils undergo pyroptosis during infection, contributing to inflammation, and are considered a primary pathological factor in conditions such as sepsis.^[^ ^142^ ^]^ Studies have revealed that sepsis in mice is associated with a substantial down‐regulation of neutrophil N‐acetyltransferase 10 (NAT10). Neutrophil‐specific NAT10 overexpression mitigates neutrophil pyroptosis and reduces sepsis‐related mortality in mice by reversing the ULK1‐STING‐NLRP3 axis.^[^ ^143^ ^]^ Pyroptosis‐induced cell death is recognized as a factor in various diseases, including cardiovascular diseases, neurological disorders, and liver conditions. A substantial portion of the ongoing research is centered on devising treatments for these diseases by targeting key inflammasome signals, such as NLRP3, Caspase‐1, or GSDMD, to inhibit pyroptosis.^[^ ^144^ ^]^

However, neutrophil pyroptosis follows non‐canonical inflammasome or pyroptotic pathways mediated by granulocyte systems. Additionally, GSDMD protein in neutrophils plays a crucial role in NET formation. The release of NETs facilitated by the lytic activity of GSDMD in neutrophils may represent a pivotal event in ARDS development.^[^ ^145^ ^]^ Consequently, blocking the GSDMD‐mediated release of NETs mediated by GSDMD might offer a promising mechanism for treating associated inflammatory diseases. Further investigations are necessary to ascertain whether neutrophil pyroptosis can be effectively targeted as a therapeutic approach.

## 5 Neutrophil Necroptosis

**Figure 6:**
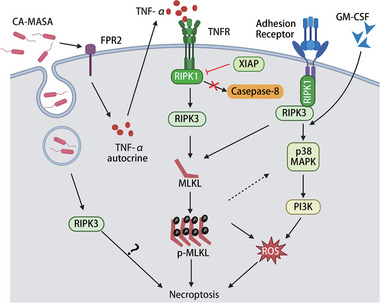
Molecular Pathways Regulating Neutrophil Necroptosis. Community‐acquired methicillin‐resistant *Staphylococcus aureus* (CA‐MRSA) is phagocytosed by neutrophils yet manages to persist intracellularly. This intracellular survival of CA‐MRSA instigates RIPK3‐mediated necroptosis, a form of programmed cell death, independently of MLKL.^[^ ^150^, ^155^ ^]^ In the absence of the XIAP, a crucial member of the IAP family, the inhibition of Caspase‐8 triggers a shift in TNF‐induced neutrophil cell death from apoptosis to RIPK1‐RIPK3‐MLKL‐dependent necroptosis.^[^ ^158^ ^]^ This transition represents a critical regulatory juncture in determining cell fate. Additionally, CA‐MRSA stimulates an autocrine production of TNFα in neutrophils, further amplifying the necroptotic cascade. In the presence of granulocyte‐macrophage colony‐stimulating factor (GM‐CSF), ligation of adhesion receptors activates the RIPK1‐RIPK3‐MLKL‐p38 MAPK‐PI3K axis, culminating in ROS production via NADPH oxidase.^[^ ^147^ ^]^ This condition is another pathway of neutrophil necroptosis. Figure created with BioRender.com.

Historically, necrosis has been considered an unregulated and spontaneous form of cell death. However, recent advancements in genetic, biochemical, and functional studies have redefined this process. Rather than being a mere accidental event, necrosis is now regarded as an active cell death pathway, termed necroptosis.^[^ ^146^ ^]^ In relation to human diseases linked to neutrophils, such as cutaneous vasculitis, ulcerative colitis, and psoriasis, neutrophils migrate to inflammation sites and activate the RIPK3‐mixed lineage kinase domain‐like (MLKL) protein pathway, leading to necroptosis (**Figure** **6**).^[^ ^147^ ^]^ Given the pivotal roles that neutrophils play in various inflammatory processes and diseases and their abundance in the body, necroptosis may play fundamental roles in the onset and perpetuation of various conditions.

### 5.1 Necroptosis in Neutrophils: Mechanisms, Regulators, and Pathophysiological Implications

Unlike extrinsic apoptosis, necroptosis operates independent of apoptotic signaling. It is predominantly orchestrated by the synergistic activity of RIPK3 and MLKL proteins.^[^ ^148^, ^149^ ^]^ Necroptosis in neutrophils is predominantly triggered by various factors, such as TNF, TLRs, granulocyte‐macrophage colony‐stimulating factor (GM‐CSF), and the engagement of adhesion receptors, such as CD44, CD11b, CD18, and CD15. Furthermore, the presence of monosodium urate crystals and phagocytosis of *Staphylococcus aureus* contribute to this process.^[^ ^147^, ^150^, ^151^ ^]^

Cellular morphological alterations during necroptosis bear striking similarities to those observed during necrosis. As neutrophils progress through necroptosis, the evident role of ROS is manifested in the degradation of organelle and azurosome membranes. This degradation sequence subsequently triggers endosomal and autophagosomal fusion, leading to the formation of expansive cytoplasmic vacuoles.^[^ ^151^ ^]^ Concomitant with organelle swelling, there is a noticeable increase in cell volume. Subsequently, the nuclear membrane ruptures, chromatin undergoes slight condensation, and the plasma membrane becomes permeable, thereby facilitating the release of intracellular DAMPs.^[^ ^152^ ^]^ Neutrophils demonstrating vacuolation undergo rapid cell death and release DAMPs in the process, thereby potentiating the pro‐inflammatory characteristics of necroptosis.

Necroptosis is initiated when the apoptotic protein Caspase is blocked. Since 2005, the significant roles of receptor‐interacting protein kinase‐1 (RIPK1) and its kinase inhibitor, necrostatin‐1, in Caspase‐independent necrosis have been reported.^[^ ^146^, ^153^ ^]^ RIPK3 has been identified as a crucial protein for necroptosis,^[^ ^148^, ^154^ ^]^ and MLKL, downstream of RIPK3, has been recognized as a necroptosis effector protein.^[^ ^149^ ^]^

Although neutrophil necroptosis has received less attention, possibly due to the short lifespan of neutrophils, a breakthrough was achieved in 2014 when Greenlee‐Wacker et al. reported that community‐acquired methicillin‐resistant *Staphylococcus aureus* (CA‐MRSA) survive after being ingested during neutrophil phagocytosis. CA‐MRSA promotes the necroptosis of neutrophils, undermines macrophage‐mediated phagocytosis of neutrophils, and exacerbates infections.^[^ ^150^ ^]^ This team subsequently ascertained that necrostatin‐1, originally thought to inhibit cell lysis, exhibits off‐target effects. Furthermore, CA‐MRSA‐phagocytic neutrophils were found to undergo a novel lytic programmed cell death driven by RIPK3 activity, independent of RIPK‐1 or MLKL activity. This form of cell death differs from traditional necroptosis.^[^ ^155^ ^]^ Some cases of necrosis may also be unrelated to RIPK1 and MLKL, as observed in myocardial necrosis induced by ischemia and oxidative stress, which act through the RIPK3‐Ca^2+^‐Calmodulin‐dependent protein kinase pathway.^[^ ^156^ ^]^ Therefore, neutrophil lysis caused by CA‐MRSA could also be interpreted as RIPK3‐dependent necroptosis (Figure 6). The ascertainment of RIPK3 involvement and unique lytic programmed cell death paradigm underscore the multifarious cell death responses elicited by CA‐MRSA.

Our understanding of necroptosis mainly stems from TNF signaling research. TNF‐triggered RIPK1‐RIPK3‐MLKL signaling pathway is the most extensively examined extracellular indicator of necroptosis (Figure 6). TNF plays a key role in initiating inflammation in response to infections and tissue damage. Within the realm of neutrophil death classification, X‐linked IAP (XIAP), an inhibitor of the IAP family, holds significant sway, dictating whether neutrophils undergo apoptosis or necroptosis. Similar to its counterpart CIAP, XIAP also ubiquitinates RIPK1, thereby extending cellular survival.^[^ ^157^ ^]^ In the absence of XIAP, Caspase inhibition prompts a shift from apoptosis to RIPK3‐ and MLKL‐dependent necroptosis. Moreover, the omission of XIAP sensitized neutrophils to TNF‐α‐induced killing.^[^ ^158^ ^]^ Research indicates that necroptosis induction can be attributed to CA‐MRSA, as it prompts neutrophils to undergo autocrine TNFα stimulation. In this context, CA‐MRSA exhibits the capacity to induce neutrophil necrosis by activating formylpeptide receptor 2‐mediated TNF‐α autocrine signaling. This activation leads to MLKL phosphorylation and increased lactate dehydrogenase release through phenol‐soluble modulins.^[^ ^159^ ^]^ (Figure 6).

Necroptosis in neutrophils, induced by the ligation of adhesion receptors and exposure to GM‐CSF, depends on the ability of NADPH oxidase to generate ROS. These ROS, in turn, activate the RIPK1‐RIPK3‐MLKL‐p38 MAPK‐PI3K signaling axis. (Figure 6).^[^ ^147^ ^]^ Remarkably, necroptosis can also occur independent of RIPK1 expression. For instance, during innate immune responses to viral and bacterial infections, Toll/IL‐1 receptor domain‐containing adaptor protein can induce IFN‐β without reliance on RIPK1. This mechanism has been observed in several studies.^[^ ^160^, ^161^ ^]^ Activation of Toll/IL‐1 receptor domain‐containing adaptor protein has emerged as a potential contributor to adhesion receptor‐triggered neutrophil necroptosis. Although the roles of p38 and PI3K in NADPH oxidase activation during neutrophil apoptosis have been documented,^[^ ^162^ ^]^ the precise interplay between the RIPK3‐MLKL complex and p38 MAPK activation remains to be fully elucidated. Notably’, in patients with chronic granulomatous disease, neutrophils fail to undergo necroptosis upon adhesion receptor stimulation,^[^ ^147^ ^]^ underscoring ROS as a crucial mediator in this form of necroptotic cell death.

### 5.2 Dual Roles of Neutrophil Necroptosis in Bacterial Infections: Protective Mechanism and Pathogenic Consequences

Neutrophils play a crucial role in eradicating pathogens through the necroptosis pathway, clearing the replication niches of pathogens, and eliminating infected cells to exert bactericidal effects. Additionally, necroptosis may contribute to the suppression of pro‐inflammatory signaling. In a *Staphylococcus aureus* infection model, wild‐type mouse neutrophils effectively eliminated bacteria at the infection site. In contrast, neutrophils lacking MLKL and human neutrophils from patients with chronic granulomatous disease exhibit diminished microorganism‐killing capabilities.^[^ ^152^, ^163^ ^]^

Nevertheless, there are instances in which bacteria can trigger neutrophil necrosis, exacerbating histopathological damage during infection. For instance, CA‐MRSA pneumonia, which is known for its drug resistance, virulence, and transmissibility, is associated with substantial morbidity and mortality. Infection scenarios can involve bacteria surviving after neutrophil phagocytosis^[^ ^164^ ^]^ disrupting initial phagocyte‐mediated defenses, and fostering persistent infection and inflammation. Neutrophil necroptosis results in the release of viable *Staphylococcus aureus* and intracellular DAMPs, thereby exacerbating the spread of infection due to localized tissue damage.^[^ ^150^ ^]^ Furthermore, CA‐MRSA can secrete virulence factors that activate MLKL phosphorylation, trigger neutrophil necroptosis, and increase lactate dehydrogenase release, ultimately causing severe lung injury.^[^ ^155^ ^]^ Regarding *Klebsiella pneumoniae* infections, neutrophil necroptosis arises not predominantly from interference with apoptotic activation, but from direct necroptosis induction. Pharmacological intervention employing RIPK1 and RIPK3 inhibitors to inhibit necroptosis‐induced efferocytosis of *Klebsiella pneumoniae*‐infected neutrophils in vitro and ameliorate disease phenotypes in mouse models of *Klebsiella pneumoniae* pneumonia.^[^ ^31^ ^]^ To conclusion, bacteria‐induced neutrophil necroptosis can be detrimental to the host infection response.

### 5.3 Neutrophil Necroptosis in Inflammatory and Autoimmune Diseases: A Double‐Edged Sword for Immunopathology and Treatment

Neutrophil necroptosis results in a substantial release of DAMPs from disrupted cell membranes. This characteristic has led to necroptosis being widely regarded as a pro‐inflammatory form of cell death. Conversely, what is frequently disregarded is that upon TNF stimulation, the majority of cells instigate a rapid and vigorous pro‐inflammatory reaction, with necroptosis potentially diminishing the production of pro‐inflammatory cytokines by curtailing neutrophil lifespan. As a result, the overall inflammatory response prompted by TNF or LPS is curtailed, ultimately restraining the inflammatory cascade.^[^ ^165^ ^]^

Characterization of the neutrophil necroptosis signaling pathway holds significant promise for the better management of tissue damage and excessive inflammation resulting from neutrophil dysfunction. This may assist in identifying suitable drug targets for neutrophil‐related diseases, including cutaneous vasculitis, ulcerative colitis, and psoriasis.^[^ ^147^ ^]^ Despite the extensive research on necroptosis, relatively little attention has been paid to its occurrence in neutrophils. The exact timing of neutrophil necroptosis remains elusive as it can transpire during migration or at sites of inflammation.

Neutrophil necroptosis plays a pivotal role in the pathogenesis of gout. In murine models of gouty arthritis induced by MSU crystals, formation akin to gout is induced through neutrophil necrosis. This process can be mitigated by downregulating RIPK3 or administering necrostatin‐1.^[^ ^166^ ^]^ In addition, studies have indicated that, in the joints of patients with rheumatoid arthritis, neutrophils activate RIPK1, RIPK3, and MLKL under the influence of CD44 and GM‐CSF, leading to necroptosis,^[^ ^167^ ^]^ thereby exacerbating tissue damage. Notably, treatment with necrostatin‐1 in murine models of rheumatoid arthritis brings in a reduction in osteoclast numbers, a decrease in Th1 and Th17 cell populations, and an increase in Th2 and Treg cell populations.^[^ ^168^ ^]^ This therapeutic approach also demonstrated the potential to decelerate disease progression.

### 5.4 Neutrophil Necroptosis and NETosis: Interplay, Inhibitors, and Implications in Immune Responses

Necroptosis in neutrophils results in the permeability of the cell plasma membrane and release of chromatin, a phenomenon potentially linked to neutrophil NETosis. Chemical inhibitors of the necroptotic pathway, such as the RIPK1 stabilizer necrostatin‐1 or MLKL inhibitor necrosulfonamide, are used to suppress necroptosis and NET release in PMA‐or monosodium urate crystal‐stimulated neutrophils.^[^ ^168^ ^]^ Classical models of NETosis involve PMA and monosodium urate crystals, which trigger ROS production, subsequently activating RIPK3, leading to phosphorylated MLKL expression. Hence, ROS is regarded as a catalyst for PMA and monosodium urate crystal‐induced necroptosis, which is intricately tied to the release of NETs.

NET liberation is an outcome or secondary event resulting from neutrophil necroptosis. However, the literature also presents conflicting evidence, suggesting that neither *RIPK3*‐deficient mouse neutrophils nor MLKL‐inhibited human neutrophils exhibit anomalous NET formation upon activation or exposure to low PMA concentrations.^[^ ^169^ ^]^ These findings indicate that NET formation is independent of RIPK3 and MLKL signal transduction. NET release during necroptosis may be the passive expulsion of chromatids associated with necrosis.^[^ ^170^ ^]^

In cases where NET formation depends on the antimicrobial response of neutrophils, necroptosis is generally perceived to have detrimental effects on the host. The precise role of neutrophil necroptosis in driving NET production remains unclear. Therefore, efficient NET detection techniques are required to differentiate between NET formation and neutrophil necroptosis.

## 6 Neutrophil Ferroptosis

Ferroptosis is a distinct type of programmed cell death characterized by iron‐mediated accumulation and peroxidation of polyunsaturated fatty acid‐containing phospholipids to lethal levels in cell membranes.^[^ ^171^ ^]^ The term “ferroptosis” was introduced by Brent R. Stockwell in 2012, highlighting the central role of glutathione peroxidase 4 (GPX4) in this process.^[^ ^172^ ^]^ Although the exploration of ferroptosis has flourished, the understanding of its impact on neutrophils remains in its infancy. Several studies have indicated that neutrophils, via mechanisms involving NETs and MPO, can trigger ferroptosis in tissues or tumor cells during inflammatory diseases, leading to adverse effects.^[^ ^173^, ^174^ ^]^

### 6.1 Neutrophil Ferroptosis: Mechanisms, Morphological Distinctions, and Regulatory Pathways

Ferroptosis is dependent on intracellular iron and is morphologically, biochemically, and genetically distinct from apoptosis, necrosis, and autophagy.^[^ ^172^ ^]^ Ferroptosis shares certain necrotic morphological traits, such as the loss of plasma membrane integrity, cytoplasmic dilation, and moderate chromatin condensation.^[^ ^175^ ^]^ Under an electron microscope, ferroptotic cells often exhibit mitochondrial anomalies, such as condensation, swelling, increased membrane density, and outer membrane rupture.^[^ ^176^, ^177^ ^]^ Neutrophils undergoing ferroptosis primarily exhibit mitochondrial dysfunction,^[^ ^178^ ^]^ with subtle morphological changes prior to cell death. During this process, the nucleus remains intact, accompanied by increased production of ROS and lipid peroxides.^[^ ^179^ ^]^

**Figure 7:**
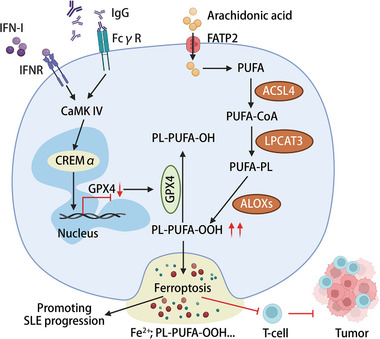
Implications of Neutrophil Ferroptosis in the Pathogenesis of Systemic Lupus Erythematosus (SLE) and Tumor Progression. In patients with SLE, autoantibodies and IFN‐α augment ferroptosis in neutrophils by intensifying the binding of the transcription suppressor cAMP response element modulator alpha (CREMα) to GPX4 promoters through activation of calcium/calmodulin kinase IV (CaMK IV), thereby reducing GPX4 expression and subsequently amplifying phospholipid‐containing polyunsaturated fatty acid hydroperoxides (PL‐PUFA‐OOH).^[^ ^178^ ^]^ Neutrophils significantly contribute to the synthesis of PUFA via the fatty acid transport protein 2 (FATP2)‐mediated uptake of arachidonic acid.^[^ ^186^ ^]^ Key enzymatic players, including acyl‐CoA synthetase long‐chain family member 4 (ACSL4), lysophosphatidylcholine acyltransferase 3 (LPCAT3), and arachidonate lipoxygenases (ALOXs), amplify the generation of PL‐PUFA‐OOH, serving as potent inducers of neutrophil ferroptosis. Neutrophil ferroptosis within the tumor microenvironment fosters tumor growth by establishing an immunosuppressive milieu, thereby impeding T‐cell‐mediated antitumor responses.^[^ ^187^ ^]^ Neutrophil ferroptosis can lead to the release of Fe^2+^ and PL‐PUFA‐OOH, thereby contributing to a range of immune regulatory mechanisms. Figure created with BioRender.com.

Ferroptosis regulation is governed by three primary pathways: (1) the system Xc‐/GSH/GPX4 pathway, where the cystine/glutamate reverse transporter (System Xc‐) comprising SLC7A11 and SLC3A2 subunits and the REDOX protection mechanism is inhibited, crucially promoting ferroptosis;^[^ ^180^ ^]^ (2) The NAD(P)H/FSP1/CoQ10 system involving FSP1 and ubiquinone (CoQ10), which capture lipid peroxyl radicals responsible for lipid peroxidation. Here, FSP1 also catalyzes the regeneration of CoQ10 using NAD(P)H, neutralizing harmful lipid peroxides;^[^ ^181^ ^]^ (3) the polyunsaturated fatty acid‐containing phospholipid synthesis system esterifies polyunsaturated fatty acids to polyunsaturated fatty acid‐containing phospholipids through ACSL4 and lipoxygenase.^[^ ^182^ ^]^ (**Figure** **7**).

### 6.2 Neutrophil Ferroptosis in Disease: Implications for Systemic Lupus Erythematosus and Tumor Microenvironment Dynamics

Neutrophils play key roles in regulating iron homeostasis and nutritional immunity during infection. Ferroptosis of neutrophils can be regarded as the result of an iron homeostasis disorder, and bacteria use free iron ions to synthesize their own substances to replicate and aggravate the infection.^[^ ^183^ ^]^ Despite advancements, the regulation of neutrophil ferroptosis in associated diseases remains relatively unexplored. Notably, ferroptosis of neutrophils has been documented in patients with SLE.^[^ ^178^ ^]^ In patients with SLE, autoantibodies and IFN‐α augment ferroptosis in neutrophils by intensifying the binding of the transcription suppressor CREMα to GPX4 promoters through activation of calcium/calmodulin kinase IV (CaMK IV), thereby reducing GPX4 expression and subsequently amplifying lipid ROS (Figure 7). Mice with neutrophil‐specific GPX4 haploinsufficiency recapitulate the key clinical features of human SLE, and treatment with ferroptosis inhibitors substantially reduces disease severity in lupus‐prone mice, underscoring the role of neutrophil ferroptosis in lupus pathogenesis.^[^ ^178^ ^]^

Iron metabolism, redox pathways, and lipid metabolism collectively regulate ferroptosis. Compelling evidence suggests that genetic and pharmacological manipulation of these pathways can effectively govern cellular ferroptosis. Given the unique attributes of neutrophils, targeting the redox pathway is pivotal for modulating ferroptosis. The System Xc–GSH–GPX4 pathway assumes a central role in ROS scavenging. Consequently, numerous drug development efforts have focused on modulating iron‐induced cell death by targeting key molecules in this pathway. The Nrf2 pathway responds to cellular oxidation by activating the transcription of genes involved in redox reactions. Targeting the KEAP1‐Nrf2 axis holds promise as a viable strategy for regulating iron‐induced cell death.^[^ ^184^ ^]^

Given their prominence in cancer research, interest in neutrophil ferroptosis within tumors has been pronounced, with this cell subset being termed myeloid‐derived suppressor cells (PMN‐MDSCs). PMN‐MDSCs exhibit a dual role, both promoting and inhibiting tumor growth, a dichotomy further emphasized by cutting‐edge single‐cell techniques that reveal neutrophil functional heterogeneity within tumors.^[^ ^185^ ^]^ Recent discoveries have revealed spontaneous ferroptosis‐driven death of PMN‐MDSCs in the tumor microenvironment, where these cells exert substantial immunosuppressive effects. In the tumor microenvironment, easily oxidized polyunsaturated fatty acid‐containing phospholipids undergo peroxidation, initiating ferroptosis and generating immunosuppressive lipid peroxidation. Both ACSL4 and FATP2 play roles in the regulation of ferroptosis and generation of immunosuppressive signals in PMN‐MDSCs.^[^ ^186^, ^187^ ^]^ The main mechanism of FATP2 mediated suppressive activity involves arachidonic acid uptake and prostaglandin E2 synthesis. Pharmacological inhibition of FATP2 can eliminate the immunosuppressive activity of PMN‐MDSCs, thereby significantly halting tumor progression. Combining FATP2 inhibition with immune checkpoint blockade has emerged as a promising strategy against tumor progression.^[^ ^186^ ^]^ In immunocompetent mice, both genetic and pharmacological ferroptosis inhibitors negate the suppressive activity of PMN‐MDSCs, thereby limiting tumor growth. Interestingly, the induction of ferroptosis appears to promote tumor expansion.^[^ ^187^ ^]^ Thus, ferroptosis is unveiled as a manipulable immunosuppressive mechanism in PMN‐MDSCs within the tumor microenvironment and presents a potential therapeutic target to counteract tumor progression.

## 7 PANoptosis: Unraveling the Multifaceted Cell Death Pathway

Although traditionally viewed as mechanistically distinct, it is now widely acknowledged that some cell death mechanisms exhibit extensive overlap. In some cases, multiple modes of cell death may coexist within one cell, leading to the conceptualization of an integrated cell death modality, known as “PANoptosis.” PANoptosis is implicated in various types of cell death, such as pyroptosis, apoptosis, and necroptosis.^[^ ^188^, ^189^ ^]^ For instance, in influenza A virus infection or TAK1 inhibition, deletion of the pyroptotic, apoptotic, or necroptotic machinery alone is insufficient and combined deficiencies of all three are necessary to prevent cell death.^[^ ^190^, ^191^, ^192^ ^]^ This genetic evidence establishes PANoptosis as a unique innate immune inflammatory programmed cell death pathway governed by a cytoplasmic multimeric protein complex, called as the PANoptosome. Malfunctions of crucial components of the PANoptosome have been linked to various human conditions, including neurodegenerative disorders, cancer, and increased susceptibility to infections.^[^ ^193^ ^]^

How the different modes of neutrophil death interplay in SLE presents a fascinating research avenue. SLE pathogenesis involves various neutrophil death modalities, with NETosis as the key player. Whether neutrophils exhibit distinct PANoptotic pathways remains unknown. Moreover, the mechanisms by which key molecules, such as MLKL and GSDMD, which are central to necroptosis and pyroptosis, influence the occurrence of NETosis remain unclear. Recent studies have revealed a significant correlation between key genes related to PANoptosis and neutrophil in patients with SLE.^[^ ^194^ ^]^ However, the occurrence of PANoptosis in neutrophils remains controversial because of the unique nature of cell death. Potential variations in the mechanism of PANoptosis‐induced cell death between neutrophils and other cell types, such as macrophages, complicate this issue. TGF‐β activated kinase 1 (TAK1), a key regulator of PANoptosis, plays contrasting roles in neutrophils and macrophages. In *TAK1*‐deficient macrophages, cytoplasmic PANoptosome assembly is increased, leading to PANoptosis. However, *TAK1*‐deficient neutrophils exhibit hyperproliferation and increased production of inflammatory cytokines.^[^ ^190^, ^195^ ^]^

A more comprehensive understanding of the molecular underpinnings of this intricate crosstalk among different cell death modalities is crucial to facilitate the development of targeted inhibitors and activators of cell death pathways as well as therapeutic interventions for diseases.

## 8 Conclusions and Future Perspectives

Neutrophils participate in various death modalities that significantly influence their antimicrobial and inflammatory responses. Given their inherent cytotoxic capabilities, stringent regulation of neutrophils is imperative to prevent unintended inflammation. Non‐lytic cell death mechanisms are predominant under homeostatic conditions. However, specific stimuli activate the lytic pathways, leading to the release of inflammatory agents and causing tissue damage. Manipulation of neutrophil death pathways may facilitate the development of novel therapies for infectious, autoimmune, and congenital neutrophil disorders.

On encountering irreparable disruptions in their intracellular or extracellular microenvironments, various death signal transduction pathways may be triggered, ultimately culminating in the death of mammalian cells. Each modality regulating cell death is initiated and perpetuated via distinct molecular mechanisms that engage in various forms of communication. Neutrophils, with their distinct functions and cell death characteristics, have a potent mechanism for ROS generation to combat the invading pathogens. ROS play diverse roles in neutrophil cell death. Therefore, ROS production and cellular localization may be critical factors linking the various neutrophil cell death pathways. Moreover, activation of neutrophils and occurrence of redox crises can result in distinct forms of cell death under various pathological conditions. In such cases, effective cellular protection may only be accomplished via interventions aimed at mitigating these crises or addressing their underlying causes rather than merely treating their manifestations.

Effective investigation of neutrophil death presents a unique challenge. Although murine models have substantially advanced our understanding of neutrophil behavior, notable differences exist between human and mouse neutrophils. These discrepancies include variations in circulating neutrophil counts, lifespan, receptor expression, and intracellular signaling pathways. These differences become especially pronounced in complex conditions, such as cancer and autoimmune disorders. Therefore, caution is advised when directly using the findings in murine models for human clinical applications. Recent development of humanized mouse models has provided a promising avenue to study human neutrophils in both health and disease conditions, thereby broadening the scope of translational research.^[^ ^196^ ^]^ Although isolating abundant pure human neutrophils from the blood is feasible, their natural propensity to undergo apoptosis post‐isolation limits their genetic manipulation. Additionally, marked heterogeneity is observed between circulating and tissue‐resident neutrophils.^[^ ^197^, ^198^ ^]^ Current endeavors are focused on establishing protocols to differentiate various stem cells into functionally active neutrophils that can aid in future research, especially genetic studies.

Although the individual death modalities have been extensively investigated, some aspects remain unknown. For example, the mechanisms by which these diverse neutrophil death pathways intersect or operate synergistically during infections remain unclear. Moreover, the feasibility of modulating these pathways to curtail the excessive release of neutrophil pro‐inflammatory mediators that exacerbate tissue injury and cause septic shock remains unknown.

## Conflict of Interest

The authors declare no conflict of interest.

## Author Contributions

Y.S. and P.L. conceived the project. H.T. and H.R. drafted the manuscript and drew the figures. All authors provided valuable comments and revised the manuscript.
